# Transcriptome Analysis of Human Glioblastoma Cells Susceptible to Infection with the Leningrad-16 Vaccine Strain of Measles Virus

**DOI:** 10.3390/v14112433

**Published:** 2022-11-02

**Authors:** Yulia Ammour, Olga Susova, George Krasnov, Eugenia Nikolaeva, Vyacheslav Varachev, Yulia Schetinina, Marina Gavrilova, Alexey Mitrofanov, Anna Poletaeva, Ali Bekyashev, Evgeny Faizuloev, Vitaly V. Zverev, Oxana A. Svitich, Tatiana V. Nasedkina

**Affiliations:** 1 I.I. Mechnikov Research Institute for Vaccines and Sera, 105064 Moscow, Russia; 2 Engelhardt Institute of Molecular Biology of the Russian Academy of Sciences, 119991 Moscow, Russia; 3 N.N. Blokhin Russian Cancer Research Center of the Ministry of Health of the Russian Federation, 115478 Moscow, Russia; 4 Department of Microbiology, Virology and Immunology of the Medical and Preventive Faculty, I.M. Sechenov First Moscow State Medical University of the Ministry of Health of the Russian Federation, 119146 Moscow, Russia

**Keywords:** oncolytic viruses, cancer immunotherapy, measles virus, glioblastoma multiforme

## Abstract

Glioblastoma multiforme (GBM) accounts for almost half of all primary malignant brain tumors in adults and has a poor prognosis. Here we demonstrated the oncolytic potential of the L-16 vaccine strain of measles virus (MV) against primary human GBM cells and characterized the genetic patterns that determine the sensitivity of primary human GBM cells to oncolytic therapy. MV replicated in all GBM cells, and seven out of eight cell lines underwent complete or partial oncolysis. RNA-Seq analysis identified about 1200 differentially expressed genes (FDR < 0.05) with at least two-fold expression level change between MV-infected and uninfected cells. Among them, the most significant upregulation was observed for interferon response, apoptosis and cytokine signaling. One out of eight GBM cell lines was defective in type I interferon production and, thus, in the post-interferon response, other cells lacked expression of different cellular defense factors. Thus, none of the cell lines displayed induction of the total gene set necessary for effective inhibition of MV replication. In the resistant cells, we detected aberrant expression of metalloproteinase genes, particularly *MMP3*. Thus, such genes could be considered intriguing candidates for further study of factors responsible for cell sensitivity and resistance to L-16 MV infection.

## 1. Introduction

Glioblastoma multiforme (GBM) is the most common and aggressive type of adult brain tumor, accounting for 55% of all brain tissue tumors [1]. The disease is invariably fatal, despite the aggressive use of multimodality treatment, with a glioblastoma cancer stem cell (CSC) population emerging as a major cause of tumor recurrence after treatment. CSCs are multipotent cells, capable of self-renewal that rapidly acquire resistance to radio- and chemo-therapeutic treatments [2]. They usually constitute 2–3% of the original glioma, but their population grows rapidly upon stimulation by surgery and treatment [3]. The current standard treatment for GBM is maximal surgery, followed by chemotherapy with temozolomide and radiotherapy [4,5]. The overall survival rate for GBM is 15 months, and the 5-year survival rate is 5–7% after the initial diagnosis. Still, nearly 90% of GBM patients will die of the disease within two years of diagnosis [6]. The lack of an efficient anti-tumor immune response and the micro-invasive nature of the glioma malignant cells have been explained by a multitude of immune-suppressive mechanisms, proven in different models. Therefore, new treatment strategies that target both the primary tumor mass and the highly resistant CSC population as well as immune suppression are imperative if GBM survival rates are to improve.

Oncolytic viruses (OVs) have been developed as an approach for the treatment of many cancers and are a particularly rational therapy choice in the treatment of tumors resistant to standard therapies. OVs not only kill the tumor cells by direct lysis [7], but the oncolytic process also provides critical danger signals in the form of virus-derived PAMPs and endogenous DAMPs as a consequence of virus-induced immunogenic cell death mechanisms, which initiate potent anti-tumor immune responses [8,9,10] to overcome the immunosuppressive tumor microenvironment [11] and induce long-lasting tumor-specific immunity. OVs have been explored as potentially new, non-cross-resistant therapeutics for the treatment of brain tumors. Safety and efficacy of herpesvirus, adenovirus, vaccinia virus, reovirus, parvovirus, poliovirus, measles virus, replicating retrovirus vector, and Newcastle disease virus (NDV) are being investigated as monotherapies [12] or in combined therapeutic regimes [13] in malignant glioma patients at different clinical phases.

The live attenuated measles virus (MV) vaccine strains have also been shown as promising therapeutic agents against human malignancies [14]. Several phase I clinical trials using the MV Edmonston-B strain have shown clinical benefits for the treatment of ovarian cancer, GBM, mesothelioma, glioma, and breast cancer [15]. Recently, it was reported that MV has significant anti-tumor activity against GBM and glioma stem cells in vitro and in vivo, a sensitivity that is at least in part explained by the overexpression of the MV receptor CD46 in brain tumor cell lines [16]. However, it is clear that elevated CD46 expression only partly accounts for viral tropism, and the anti-viral state of the host likely plays an important role in susceptibility to MV [17].

In the present study, the oncolytic properties of MV have been investigated in vitro against primary GBM cells. In our study, we focused on the attenuated Russian vaccine strain of MV, “Leningrad-16” (L-16), which has been recently evaluated as an OV against human melanoma cells [18]. L-16 was isolated in rodent guinea-pig kidney cell culture after 17 passages at 35–36 °C followed by four passages in the same cell culture at 30 °C and 7 passages in Japanese quail embryo fibroblast cells at 35–36 °C. Such adaptation of the L-16 strain has provided intensive viral reproduction. As Edmonston-B MV, L-16 has an excellent safety record as a human vaccine strain [19].

To better characterize MV-induced immunological responses at a transcriptional level, mRNA sequencing was performed to examine gene expression in infected primary GBM cells. Possible predictive gene changes in GBM cells sensitive to L-16 MV-induced oncolysis were determined.

## 2. Materials and Methods

### 2.1. Cell Cultures, Virus and Viral Propagation

Glioblastoma (GBM) cell lines Gbl7n, Gbl11n, Gbl12n, Gbl13n, Gbl16n, Gbl17n, Gbl24n, and Gbl25n at early passages (6–7 passages) were established from primary tumor cells in the N.N. Blokhin Russian Cancer Research Center from surgical resection or biopsies of confirmed GBM cases and genetically characterized. All patients gave their informed consent. The molecular–genetic profile of cell lines was performed using next-generation sequencing [20]. The mutations found are summarized in Table 1. Immortalized human epithelial cells, hTERT-NKE (normal kidney epithelial cells), were obtained from the collection of the N.N. Blokhin Russian Cancer Research Center.

The L-16 vaccine strain of MV, obtained from the collection of the I.I. Mechnikov Research Institute for Vaccines and Sera (titer 6.2–6.3 log_10_ CCID_50_/mL), was propagated in Vero (CCL-81, ATCC, USA) and estimated by the end-point dilution CCID_50_ assays in Vero cells.

All cell lines were maintained in T25 flasks (Corning, Amsterdam, Netherlands) in the DMEM/F12 culture medium (Thermo Fisher Scientific, Munich, Germany) supplemented with 10 % fetal bovine serum (FBS; Thermo Fisher Scientific, Munich, Germany), 1 mM glutamine (Thermo Fisher Scientific, Munich, Germany) and 50 μg/mL gentamicin reagent solution (Thermo Fisher Scientific, Munich, Germany) in a humidified atmosphere under 5 % CO_2_ at 37 °C. Cultures were passaged once confluent. Cells were split with TrypLE Express (Thermo Fisher Scientific, Munich, Germany). To assess the cell viability, a cell suspension was supplemented with 0.2% trypan blue (Thermo Fisher Scientific, Munich, Germany), and cells were counted in a Goryaev chamber.

To perform infection of GBM cells, 80–90 % confluent cell monolayers were washed once with 3 mL of Hanks’ Balanced Salt Solution (HBSS, Thermo Fisher Scientific, Munich, Germany) prior to infection. Virus stock suspension was diluted in an FBS-free culture medium and then used to infect cells at a multiplicity of infection (MOI) of 1.0 or 0.01 infectious units per cell; cells were incubated at 32 ± 1 °C in 5 % CO_2_ for 3 h. Mock-infected control cultures were processed in parallel with MV inactivated by exposure to UV light (20,000 μJ/sm^2^, 20 min on ice). Following virus adsorption, the inoculum was removed, the cell monolayers were washed three times with HBSS and cultured in DMEM/F12 medium supplemented with 2 % FBS in an atmosphere containing 5 % CO_2_ at 32 ± 1 °C for 5–7 days. To study the virus accumulation kinetics, supernatants were collected at the indicated time points, clarified by centrifugation at 3370× *g* for 10 min to remove debris, and stored at −70 °C until testing. RNA samples isolated from three independently MV-infected or mock-infected cell cultures were used for each analysis. The amount of intracellular virus was determined by dissolving the cell pellet in 500 μL fresh medium followed by three subsequent freeze–thaw cycles and clarified by centrifugation as described above. Virus titers were determined by the end-point dilution CCID_50_ assay on Vero cells in duplicate.

For high throughput analysis, 1.0 × 10^6^ cells were infected with MV at a MOI of 1.0 or mock-infected. Cells were lysed with 300 μL RLT buffer (RNeasy kit, Qiagen, Hilden, Germany) in duplicates at 36 h post infection and stored at –70 °C until use.

### 2.2. Viral and Total RNA Extraction

Viral RNA was isolated from cell culture supernatants using the QIAamp Viral RNA Mini Kit (Qiagen, Hilden, Germany) from 140 μL of the virus-containing supernatant, while total RNA was isolated from cell lysates in RLT buffer using the RNeasy kit (Qiagen, Hilden, Germany) according to the manufacturer’s instruction. Total RNA was quantified using a NanoDrop^®^ ND-1000 spectrophotometer (NanoDrop ND-Thermo Fisher Scientific, Munich, Germany). RNA quality and quantity were assessed using the 2100-Bioanalyzer (Agilent Technologies, Waldronn, Germany). RIN index (RNA integrity index) was 9.5–10.

### 2.3. Quantitative Reverse Transcription PCR (RT-qPCR)

Viral RNA quantification was performed using real-time RT-qPCR as described previously [21]. Thermal cycling was performed in the DT-Prime5 (DNA-Technology, Moscow, Russia) instrument. The cycling conditions included 95 °C for 120 s, 45 cycles of 58 °C for 50 s, and 95 °C for 20 s.

Each sample was tested in duplicate. The output of the RT-qPCR for each sample was the threshold cycle (C_t_) value measured by the second derivative maximum method of the instrument software. In parallel with samples a 10-fold dilution series of reference MV with known titers (expressed in log_10_ CCID_50_/mL) was run in duplicate to construct a 4-point calibration curve. Thus, titers for the test samples were calculated in CCID_50_/mL relative to reference preparations based on the standard curve and subsequently converted to the log_10_CCID_50_/mL values.

### 2.4. RNA Sequencing

Libraries for sequencing were prepared using the TruSeqRNA Library Preparation Kit v2 (Illumina, San Diego, CA, USA). About 1–2 µg of total isolated RNA was used for each sample. The final size of the cDNA library fragments was 400–500 bp. To accurately determine the concentration of the obtained cDNA, quantitative PCR was used, then a mixture of cDNA libraries of different samples was prepared in equal proportions, and the mixed libraries were validated. Sequencing was performed on the NextSeq500 (Illumina, San Diego, CA, USA) platform in the pair-end read mode (2 × 150 bp). There were two runs of NextSeq500: 12 samples per run. For each sample, about 30–50 million reads were obtained.

The derived reads were checked up with FastQC 0.11.5, trimmed with Trimmomatic 0.39 and then mapped to the human reference genome GRCh38 (Ensembl version 102 annotation) using STAR 2.7.5a. Read counts per gene were estimated using the featureCounts tool from the Subread 1.6.0 package. Additionally, we ensured the absence of 3′-bias with the geneBody_coverage script from the RSeQC 3.0.1 toolkit. Differential gene expression analysis was performed in an R environment using the edgeR 3.28 package. Gene Ontology, KEGG, Reactome, and WikiPathways enrichment analyses were made up with clusterProfiler 3.14.3 and topGO 2.38 packages. Pathway visualization was performed with pathview 1.26. GO-centric profiles were created with in-house-developed scripts.

### 2.5. Viability Assay

1.5 × 10^4^ cells per well were seeded into 96-well culture plates for 12 h followed by MV-infection using a virus-containing supernatant as described above at various MOI values and incubated in an atmosphere containing 5% CO_2_ at 32 °C for 3–96 h. At various times after infection, 3-(4,5-dimethylthiazol-2-yl)-2,5-diphenyltetrazolium bromide (MTT, Promega, Madison, WI, USA) reagent was added at 5 μg/mL per well, and cells were then incubated further for 3 h at 32 ± 1 °C in 5 % CO_2_. The reaction was stopped by washing the cell monolayer and adding 60 μL dimethyl sulfoxide (DMSO, Sigma-Aldrich, USA) solution to each well. Plates were shaken at 300 rpm for 10 min to provide for a homogeneous dye distribution. The optical density (OD) was measured at wavelengths of 570 and 630 nm using a microplate reader (Varioskan Flash, Thermo Fisher Scientific, Munich, Germany). The OD for an infected cell culture in an indicating time point was expressed as a percentage of OD obtained for this cell culture at 3 h post-infection. Results were described as mean values, which were measured in 12 replicates and repeated from three independent experiments. The EC_50_ was defined as the inhibitory amount of virus that reduced the absorbance of treated infected cells to 50% when compared with that of mock-infected cells.

In parallel, at 48 h after virus inoculation, the Caspase-Glo^®^ 3/7 reagent (Promega, Madison, WI, USA) was added in 100 μL to each well according to the manufacturer’s instruction. The luminescence of each sample was measured using Varioskan Flash microplate reader.

### 2.6. Flow Cytometry

A total of 1.0 × 10^5^ cells were incubated with FITC-conjugated anti-human CD46 antibodies (MEM-258 clone, BioLegend, London, UK) or isotype control FITC-conjugated mouse IgG1 (BioLegend, London, UK) in PBS containing 1.0% FBS and stained at 4 °C for 30 min. Then, cells were washed twice, resuspended in PBS containing 1.0% FBS and fixed with 1 % paraformaldehyde (PFA, Sigma-Aldrich, Darmstadt, Germany) for 30 min at 4 °C. Analysis by flow cytometry was performed using the EPICS XL (Beckman Coulter, Brea, CA, USA) flow cytometer and the software SYSTEM II (Beckman Coulter, Brea, CA, USA).

### 2.7. ELISA

The level of TNFα, IL-6, IL-1β, and IL-10 was measured in cell culture supernatants using the TNFα-IFA-BEST, IL6-IFA-BEST, IL1β-IFA-BEST, and IL10-IFA-BEST kits, respectively, according to manufacturer’s instruction (Vector-best, Novosibirsk, Russia).

### 2.8. Statistical Analysis

The significance of the difference between experimental and control groups was analyzed using a two-tailed Student’s *t*-test. Estimating effective doses, 50% effective concentration (EC_50_), was conducted with asymptotic-based confidence intervals. Data were expressed as mean ± standard deviation (SD). A *p*-value of <0.05 was considered statically significant.

## 3. Results

### 3.1. Glioblastoma Cells Are Susceptible to the Oncolysis Induced by the MV L-16 Strain

The susceptibility of human GBM cells to virus-mediated oncolysis was tested in a panel of eight cell lines derived from patients. To prove the origin of established cell lines, GBM cells were preliminarily characterized by marker proteins of neuroectodermal stem cells (Supplementary Figure S1) and mutation profiling (Table 1).

Eight human primary GBM cells (Gbl7n, Gbl11n, Gbl12n, Gbl13n, Gbl16n, Gbl17n, Gbl24n, and Gbl25n) were infected with 10-fold serial dilutions of an L-16 strain of MV and the infection was followed through 96 h to analyze elimination of tumor cells. Immortalized human non-tumor kidney epithelial hTERT-NKE (NKE) cells were used as a control to determine virus specificity for GBM cells. NKE cells were chosen due to the rapid proliferative rate such as doubling time was comparable to that of the majority of GBM cells used in this study.

**Figure 1:**
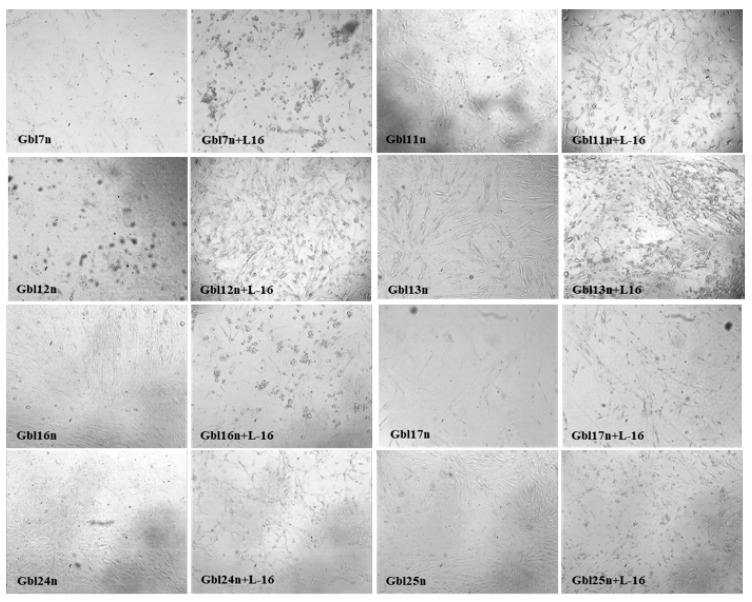
Cytopathic effect of MV (strain L-16) on Gbl7n, Gbl11n, Gbl13n, Gbl16n, Gbl17n, Gbl24n, and Gbl25n cells. The figure shows photographs of cell lines at 48 h.p.i. at an MOI 1.0; control cells were incubated with UV-inactivated virus. Magnification 10×.

The cytopathic effects (CPE) in MV infected GBM cells were visible at 48–72 h post-infection (h.p.i.) representing the granulation in the cytoplasm, rounding and progressive detachment of cells. The distinctive MV-induced CPE was detectable in all GBM cell lines but not in non-tumor NKE cells. The CPE correlated with a used MOI, becoming more obvious over time (Figure 1). It should be noted that the Gbl12n cell line, with much slower CPE development induced by MV infection (as these cells were not resistant to mumps virus induced oncolysis), differs from other used GBM cell lines with much slower growth (longer doubling time).

**Figure 2:**
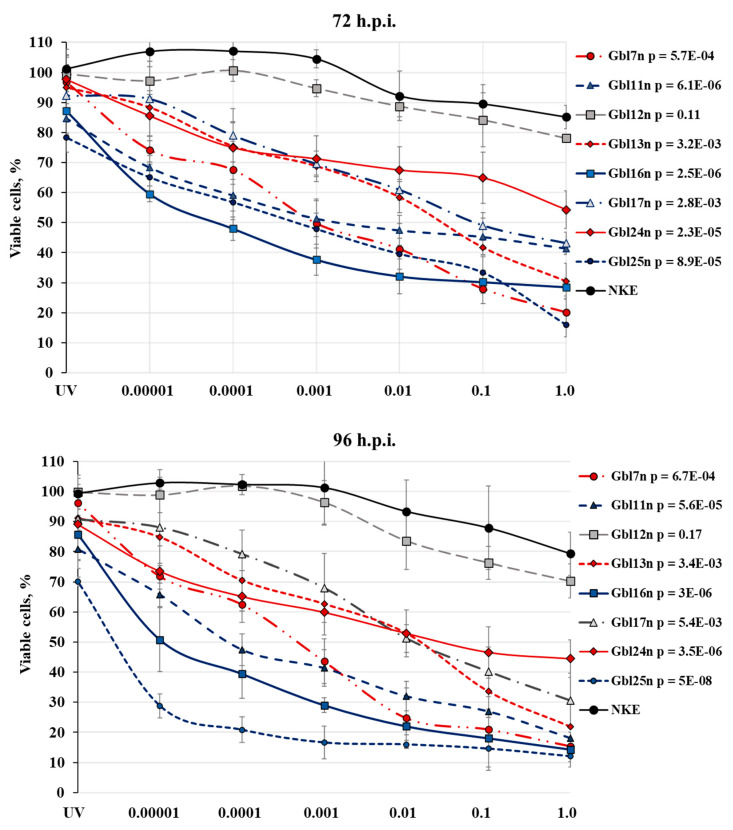
Oncolytic activity of MV strain L-16 against human GBM cell lines at 72 and 96 h.p.i. The viability of each cell line infected with MV at an MOI indicated on horizontal axes was measured using an MTT assay; cells treated with UV-inactivated MV were used as control. The data shown are mean results from three independent experiments. Horizontal axis represents the % of viability of treated cells at indicated time points; cell viability at 3 h.p.i. is taken as 100%. Bars demonstrate standard deviation (SD); calculated p-values (MV infected NKE versus MV infected GBM cells) are shown. Average CV composed from 0.41% to 15.63%.

Cell survival was assessed by measuring the mitochondrial activity using the MTT assay and expressed in percentage for an indicated time point during 96 h experiment (Figure 2). The GBM cell lines showed a broad range of sensitivities to MV-induced oncolysis. Four out of eight GBM cell lines, Gbl7n; Gbl11n; Gbl16n; and Gbl25n, were shown to be susceptible to MV oncolysis at MOIs of 1.0 to 0.001 (*p* < 0.001), with a Gbl25n being the most susceptible even at a MOI of 0.00001. Thus, at 96 h after infection of Gbl7n; Gbl11n; Gbl16n; and Gbl25n cell lines, a proportion of reminding viable cells composed from 15.4; 18.2; 14.3; and 12.0 % at a MOI of 1.0 to 43.6; 41.4; 29.0; and 16.6 % at a MOI of 0.001, respectively, relative to the corresponding uninfected cell line. Three GBM cell lines, Gbl13n; Gbl17n; and Gbl24n, were relatively susceptible at MOI of 1.0 to 0.001 (*p* < 0.01): a proportion of reminding viable cells composed of 21.9; 30.5; and 44.5 % at a MOI of 1.0 and 62.7, 67.9, and 59.9 % at a MOI of 0.001, respectively. Consistent with the lack of visible CPE development, the cytotoxic effect of the MV-infection was least pronounced for Gbl12n and did not reach statistical significance (*p* > 0.05); the pattern of reminding viable cells in the dose–response curve for the Gbl12n was similar to the NKE cells used as the control (Figure 2) line and composed of 70.3 and 79.3 % at a MOI of 1.0 and 83.6 and 93.4 % at a MOI of 0.01, respectively, at 96 h post infection.

Using obtained cell viability dose–response curves, the EC_50_ dilution of MV for each cell line was calculated with high statistical reliability (R2 > 0.97), indicating EC_50_ dilution of input MV as 2.60; 2.18; 1.37; and 0.56 log_10_ CCID_50_/mL for Gbl7n; Gbl11n; Gbl16n; and Gbl25n cell lines, respectively; 4.11; 4.36; and 4.7 log_10_ CCID_50_/mL—Gbl13n; Gbl17n; and Gbl24n cell lines, respectively; while for Gbl12n and NKE cell lines it was >6.2 log_10_ CCID_50_/mL.

**Figure 3:**
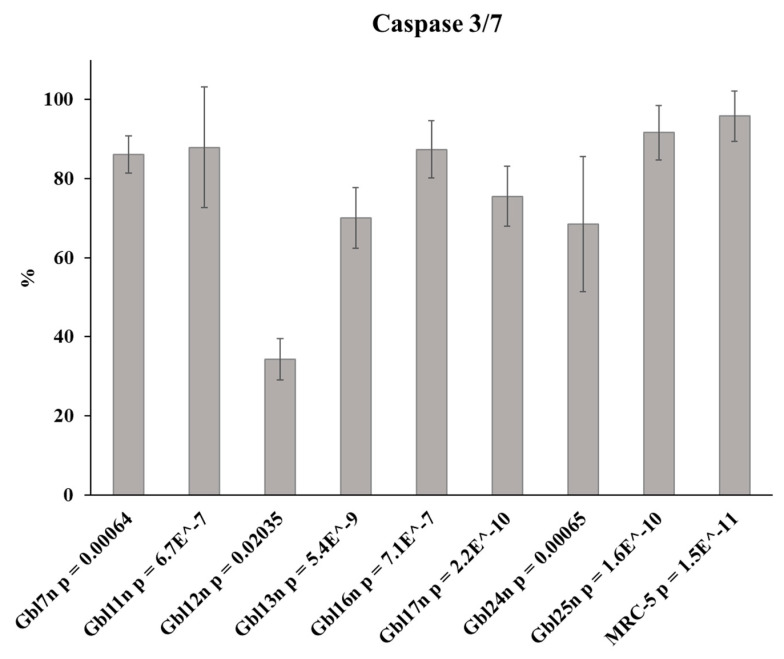
Caspase-3 and -7 activity induction in MV infected GBM cells and MRC-5 control cells. GBM or MRC-5 cells were infected with MV at a MOI of 1.0, and the level of caspases’ activity was measured and expressed as a percentage of the difference between mock-infected and MV-infected GBM cells. The data shown are mean results from three independent experiments. Bars demonstrate SD, calculated *p*-values (mock-infected versus MV-infected) are shown.

Analysis of the activity of proapoptotic caspases in MV-infected cells revealed a significant increase in the activity of caspase 3 and 7 (*p* < 0.05) in all GBM cell lines at 48 h.p.i., including Gbl12n (Figure 3). To control the induction of caspases’ activity, human fetal fibroblasts, MRC-5, were used as these cells are permissive to vaccine strains of the MV, including L-16. As expected, activation of caspases three and seven were observed for these control cells. These results demonstrate that caspases activate in all GBM cells in response to MV infection. The highest values of the caspases’ activity were observed in Gbl7n, Gbl11n, Gbl16n, and Gbl25n cells, while in Gbl13n, Gbl17n, and Gbl24n the caspases’ activation was lower, with Gbl12n showing minimal caspases’ activation which, in turn, showed resistance to MV-induced CPE and oncolysis. Thus, the caspase-3 and -7 activation profiles correlated with MTT results.

### 3.2. MV L-16 Strain Replicates in Human Glioblastoma Cells

**Figure 4:**
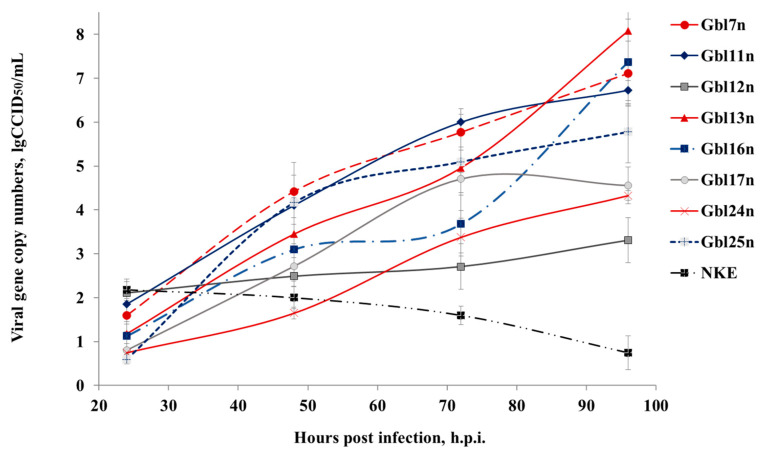
MV RNA accumulates in supernatants of infected GBM cells. GBM and NKE cells were infected with MV at a MOI of 0.1 and viral RNA released to the supernatants was quantified. Data shown represent the mean of three independent experiments and expressed in CCID_50_/mL calculated relative to the calibrator sample with known titer. Error bands indicate SD.

**Figure 5:**
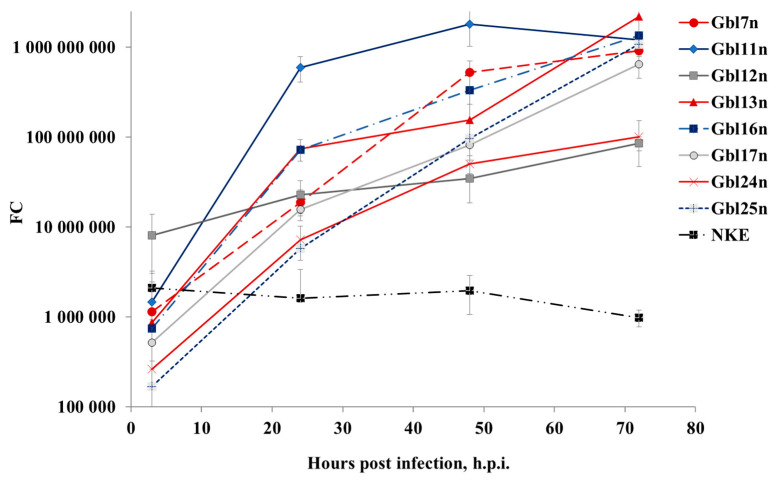
MV RNAs accumulate in infected GBM cells. GBM and NKE cells were infected with MV at a MOI of 0.1 and virus RNAs were quantified in cell lysates using the RT-qPCR method. The data shown are representative of three independent experiments. Vertical axes show fold change FC; average values of threshold cycles (Ct) for MV intracellular RNA obtained for each sample normalized to average Ct values for house-keeping genes: *GAPDH* and *ACTB*, measured for the same sample in parallel (∆Ct)). The calculation was performed relatively to normalized Ct values for the respective mock-infected cell culture 3 h.p.i. (∆∆Ct). Data represent the FC values between the level of MV RNA expression for MV-infected (∆∆Ct(+)) and mock-infected (∆∆Ct(−)) cell lines for each time point. Error bands indicate SD.

Since it has been shown that different GBM cells varied in viral oncolysis observed, the cells were infected with MV at a MOI of 0.1 and both intracellular MV replication and extracellular viral RNA accumulation were measured by real-time RT-qPCR. It was observed that overall the efficiency of viral RNA replication in all cell lines tested correlated with the kinetics of reduction of cell viability. Viral RNAs accumulated within 96 h of cultivating period both in the supernatants (Figure 4) and lysates (Figure 5) of infected cell lines. In supernatants, the MV titer significantly increased up to 72 h.p.i. for Gbl11n, Gbl17n, and Gbl25n cells after which plateau levels were reached. For Gbl13n and Gbl16n cell lines, titers continued to grow during the course of the experiment reaching the highest values at 96 h.p.i. Titers in supernatants of infected Gbl12n cells remained almost constant during the observation period and no virus replication was observed for control NKE cells.

Similar trends were observed for amounts of viral RNA in cell lysates: RNA amounts hugely increased during the course of infection for all used GBM cell lines, except for the Gbl12n cell line where only a minimal increase was observed. It should also be noted that the lowest values of the virus titer and viral RNA concentration were observed for the Gbl12n and Gbl24n cell cultures.

Taken together, obtained data indicates that all our GBM cell lines were permissive for MV infection. This contrasts to the NKE cell line for which a decrease of viral RNA concentration both in supernatants and cell lysates was observed indicating that these cells are resistant to MV infection.

**Figure 6:**
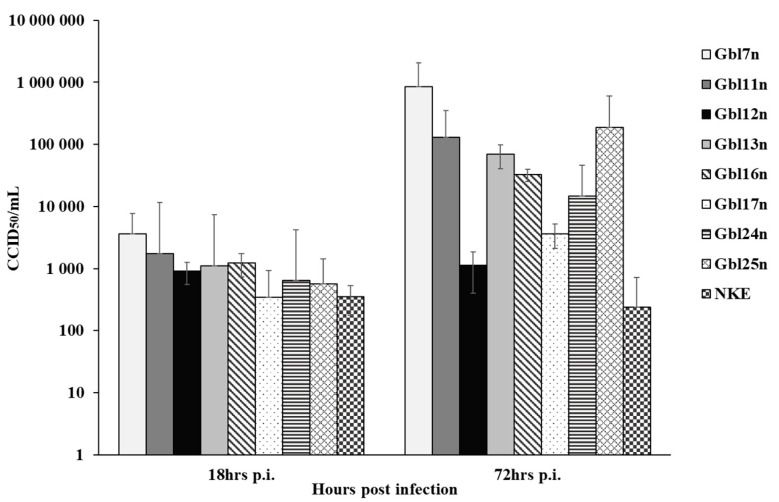
GBM cell-associated MV titers at 18 and 72 h p.i. GBM and NKE cells were infected with MV at a MOI of 0.1 and virus titers were determined by the end-point dilution CCID_50_ assay on Vero cells in duplicate. Error bands indicate SD. Vertical axes expressed in logarithmic scale.

To investigate if GBM cells are synchronously infected, specifically if cell populations are infected to the same level across all GBM cell used, the infectious virus yield from infected GBM cells were examined using the end-point dilution CCID_50_ assay at 18 and 72 h.p.i. Similar rates of primary infection were observed for Gbl11n, Gbl13n, and Gbl16n cells infected with different MOIs, 1749.48 CCID_50_/mL, 1106.63 CCID_50_/mL, and 1231.73 CCID_50_/mL, respectively. The MV infection of Gbl12n, Gbl24n, and Gbl25n cells produced slightly lower viral titers, 906.77 CCID_50_/mL, 645.65 CCID_50_/mL, and 561.68 CCID_50_/mL, respectively; while Gbl7n cells showed the highest titer at 18 h.p.i., 3633.75 CCID_50_/mL, and Gbl17n cells—the lowest, 346.52 CCID_50_/mL, (Figure 6). Titer growth at 72 h.p.i. showed that MV effectively replicates in GBM cells, except for Gbl12n. The highest titers at this time point were reached for Gbl7n, Gbl11n, and Gbl25n cells—5.93, 5.11 and 5.27 log_10_CCID_50_/mL respectively, while the lowest titers were observed for Gbl12n, Gbl17n, and Gbl24n cells, 3.05, 3.56 and 4.34 log_10_CCID_50_/mL respectively, in agreement with MV RNA replication patterns.

Together, these data indicate that differences in viral replication in GBM cells are not caused by differences in viral entry but rather are due to subsequent inhibition of MV replication.

### 3.3. Human Glioblastoma Cells Express the MV Receptor

**Figure 7:**
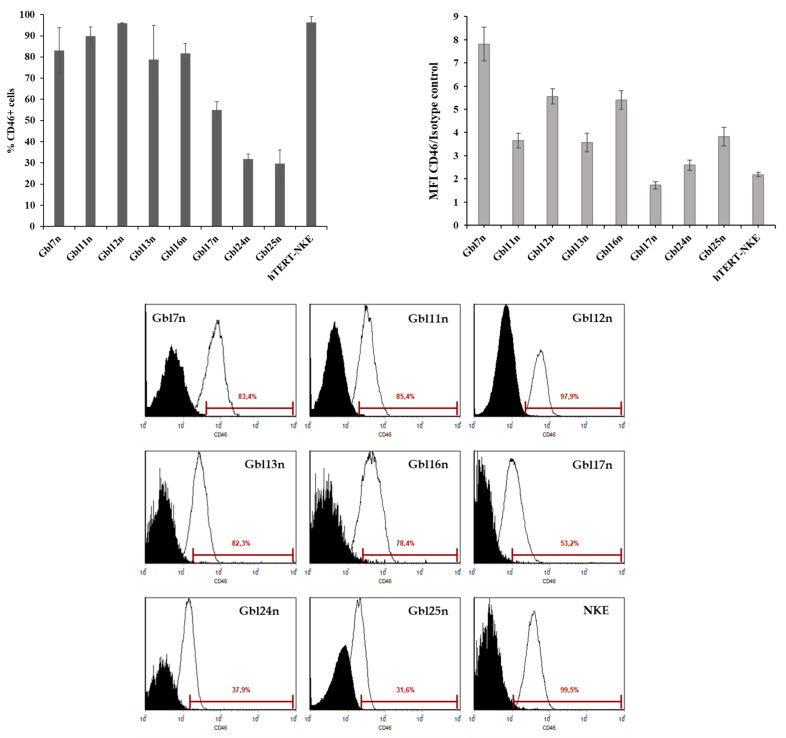
Expression of the CD46 receptor at the surface of glioblastoma cells and NKE as measured by flow cytometry. A total of 1.0 × 10^5^ cells were incubated with a FITC-conjugated anti-human CD46 antibodies and analyzed using the EPICS XL (Beckman Coulter) flow cytometer for expression levels of CD46 by determining mean fluorescence intensity (MFI) normalized to isotype control using the SYSTEM II software (Beckman Coulter). Data shown are mean results from three separate experiments; bars demonstrate SD. Shaded histograms represent isotype control staining; solid line histograms represent CD46 staining.

Although there are several receptors required for the attachment of MV to host cells, previous studies have identified CD46 as playing a key role for the MV L-16 [18]. Furthermore, CD46 molecules playing the role of the receptor for attenuated strains of MV are known to be upregulated on the surface of tumor cells [22]. As the presence of CD46 molecules may affect the efficiency of MV-induced oncolysis, the expression level of CD46 on the surface of GBM and NKE cell lines was measured by flow cytometry. The expression of CD46 was detected on a surface of approximately 30 % to 96 % of GBM cells and nearly all NKE cells (Figure 7). However, the level of CD46 expression determining by relative mean fluorescence intensity was 1.5–3.5 times higher for GBM cells compared to NKE cells, except for Gbl17n cells with the lowest CD46 expression and Gbl24n cells with comparable CD46 expression. Thus, no significant correlation between different sensitivity of cell lines toward MV oncolysis and expression of CD46 was observed (compare Figure 2 and Figure 7) suggesting that CD46 expression was necessary but did not play a role as a key factor of MV selectivity to GBM cells. Thus, other factors should be implicated in the different sensitivity of GBM cell lines to MV oncolysis.

### 3.4. Transcriptome Analysis in MV-Infected Glioblastoma Cells Reveals Type I Interferon Pathway Activation

As other studies have previously reported that many cancer cells are defective in type I interferon (IFN) signaling [23], the whole transcriptome analysis was performed for GBM cell lines. In order to insure a relatively high number of infected GBM cells and avoid asynchronous infection due to spreading viral progeny, cells were infected at a MOI of 1.0 and analysis of host cell transcription was performed at 36 h.p.i. to avoid multiple viral life cycles.

**Figure 8:**
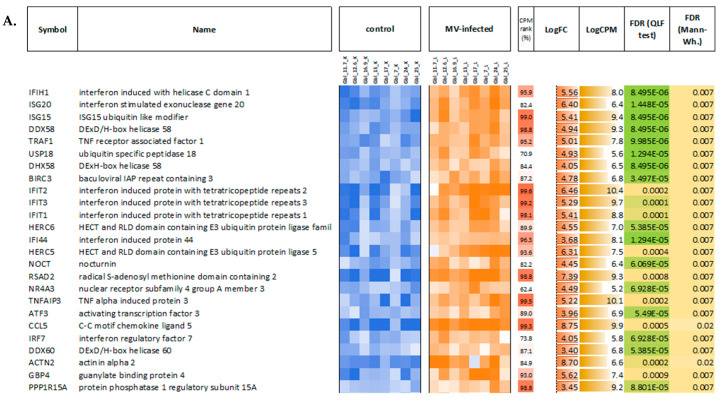

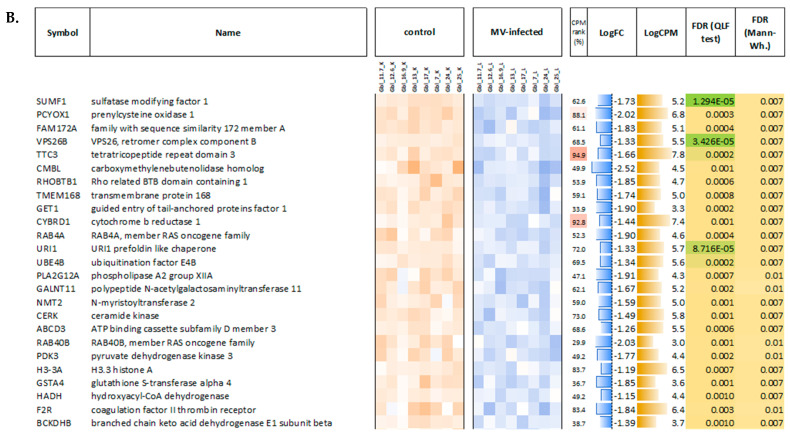
Top 25 upregulated (**A**) and downregulated (**B**) genes in MV-infected GBM cells compared to the mock-infected control ones. CPM tank (%)—position of a gene (0…100) in a list of all genes sorted by average expression level (read counts per million). LogFC—binary logarithm of expression level fold change. QLF—quasi-likelihood F-test. Mann–Wh.—non-parametric Mann–Whitney U-test. Control, MV-infected—mini heatmaps illustrating per-sample gene expression profiles normalized to the average value (geometric mean) across all samples, per each gene (blue-to-red color logarithmic scale; from 16-fold downregulated to 16-fold upregulated relative to the average values).

First, the obtained data on MV-infected and mock-infected GBM cells were compared to each other and between different GBM cell lines to study the responses to MV infections and to determine the relative modulation of cellular transcription induced by MV infection. When comparing pools of MV-infected and non-infected GBM cells, a total of 1196 genes demonstrated statistically significant differential expression (FDR < 0.05) with at least a two-fold expression level change. Most of them (859 genes) were upregulated, and only 337 were downregulated in MV-infected cells. As seen in Figure 8, genes that are upregulated in MV-infected cells have very high expression levels (see the “CPM rank” column in Figure 8).

**Figure 9:**
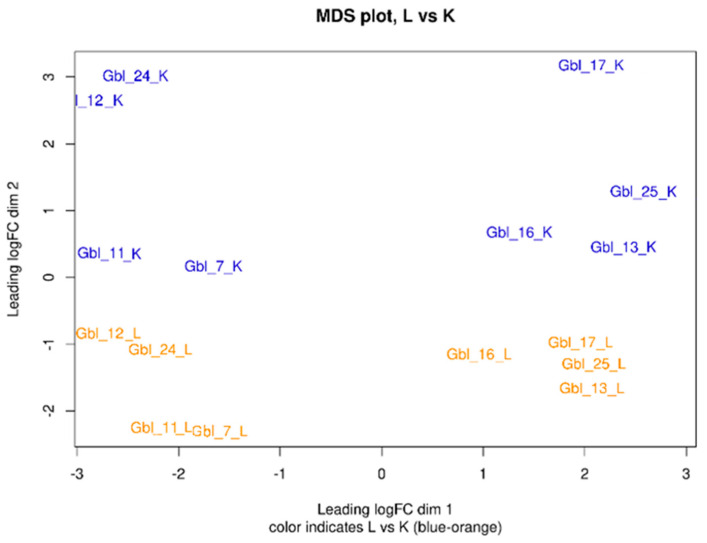
Multidimensional scaling (MDS) plot describing differences in gene expression profiles between MV-infected (orange) and control (blue) GBM cell lines. K—mock-infected GBM cells; L—MV-infected GBM cells. The numbers in brackets denotes the percentage of variance explained. Thus, the split on the basis of virus infection falls on the 2nd dimension. The 1st dimension split cell lines on the basis of the presence of mutations in the *PTEN* gene (or both the *PTEN* and *TP53* genes). Thus, cell lines carrying mutations in these genes (Gbl13n, Gbl16n, Gbl17n, and Gbl25n) appeared on the right.

Multidimensional scaling (MDS) analysis based on correlation of gene expression among samples (distance was calculated as “1—Pearson correlation coefficient” for log-transformed gene expression values, i.e., CPMs) for top-500 variable genes ensured that the biological variability of individual cell lines did not overcome the differences between groups (MV-infected, mock-infected control) to compare, which was completely described with dimension 2 (see MDS plot on Figure 9). Hierarchical clustering revealed the similarity among the commonly changed genes. Gbl12n and Gbl24n cells, mock-infected as well as infected, were clustering relatively close. Another clustering group was composed of Gbl7n and Gbl11n cells. Furthermore, all *PTEN*-mutated cells: Gbl13n, Gbl16n, Gbl17n, and Gbl25n, mock-infected as well as MV-infected, were clustering together on the one side of the MDS plot. Among this group of *TP53*-mutated cells, Gbl13n, Gbl16n, and Gbl25n were sub-clustering relatively closer. In all GBM cells, MV infection shortened the distance between groups.

In general, the cellular response to infection in different cell lines (including sensitive and resistant ones) was very similar in a variety of biological processes and pathways. Overall, the main difference was revealed in the amplitude of these changes. Cardinal differences in gene expression profiles between cell lines were noted only in individual cellular pathways.

**Figure 10:**
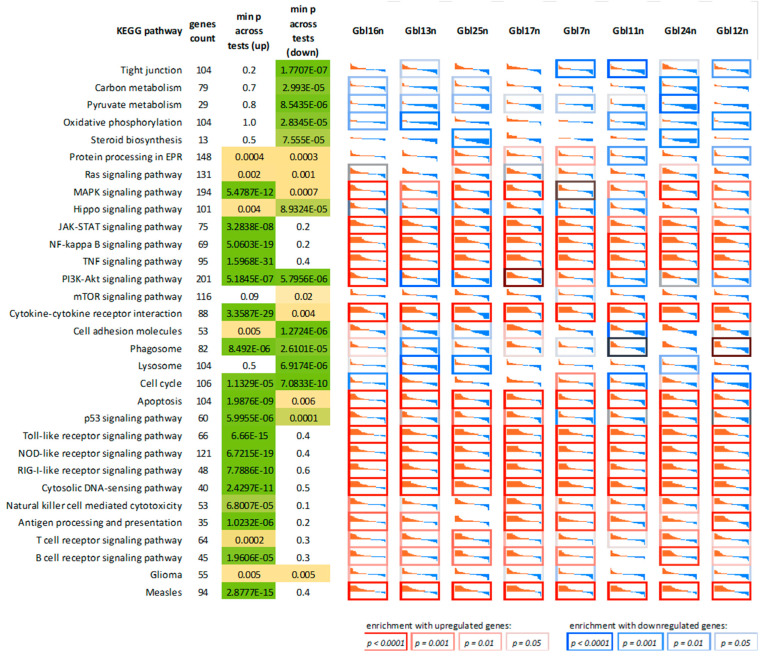
Relative gene expression profiles for genes participating in KEGG pathways that are mostly affected by MV infection, as well as several additional pathways. In each cell, there are shown sorted gene expression fold changes, from most upregulated (red) to downregulated (blue). Gene expression values are log2-transformed. In each cell, vertical axis limits are a four-fold increase (red) to a four-fold expression level decrease (blue). The cell borders demonstrate gene set enrichment *p*-value.

**Figure 11:**
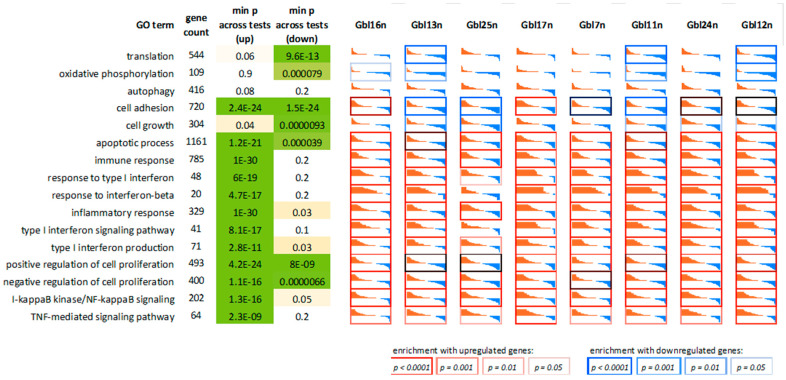
Relative gene expression profiles for genes involved in several significant biological processes (GO terms), some of which were not covered by the KEGG database (previous figure), including interferon response.

**Figure 12:**
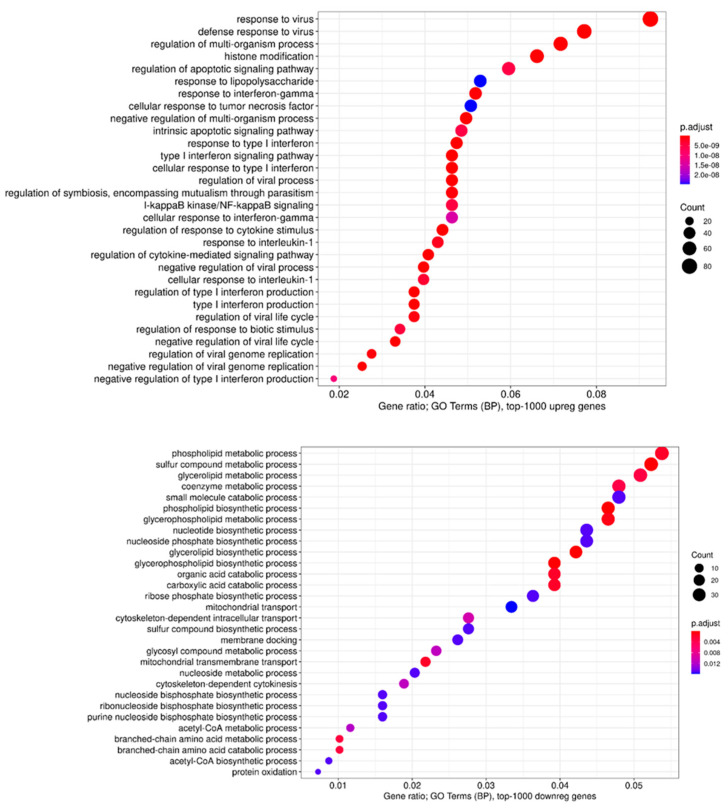
Dot plot for enriched GO terms (biological processes) for top 1000 upregulated genes (upper panel) and 1000 downregulated genes (down panel) in MV-infected cells compared to uninfected cells. The horizontal axis indicates gene ratio—a proportion of genes in a term that are differentially expressed. Bubble colors indicate adjusted enrichment *p*-value (FDR). Bubble sizes indicate counts of differentially expressed genes in a term.

Next, we performed GO terms, KEGG, Reactome, and WikiPathways enrichment analyses for lists of the top 50, 100, 200, and 500 differentially expressed genes (DEGs) (Figure 10 and Figure 11, and summarized in Figure 12). As expected, we found a variety of immune system-related processes enriched with upregulated genes: immune response (IR), including IR to a virus, innate IR, production of interferon-α/β/γ (incl. DDX58/IFIH1-mediated), and response to interferons, RIG-I, TLR, and NF-κB (incl. TRAF6-mediated) pathways, cytokine production and response to cytokines, cellular response to a virus, inflammatory response, production of TNF and response to TNFs, viral replication. The spectrum of enriched pathways for downregulated DEGs was less focused. Downregulation has been established for genes involved in dozens of metabolic processes, endocytosis, regulation of transcription, and mRNA splicing.

The GO terms involved in cellular defense mechanisms to the virus were the most enriched in all infected cells. *IFNB1*, *IFNL1*, *IFNL2*, *IFNL3*, *RSAD2*, *OASL*, *IFIT1*, *IFIT2*, *IFIT3*, *HERC5*, *MX1*, *OAS1*, *IFITM1*, *IFIH1*, *DDX58*, *MMP12*, *ADAR*, and other IFN and immune response genes were among the most upregulated genes participating in GO term GO:0051607 (defense response to the virus). Furthermore, constitutive activation of the type I IFN signaling was the second most differentially activated pathway (GO:0060337). Among the total identified 73 IFN response genes in the genome, the same genes cited above were the most upregulated. Downregulated genes included *RNASEL, ABCE1, PTPN11, MAVS*, and *CNOT7*. Negative regulation of viral genome replication (GO:0045071) was also highlighted including the most upregulated genes such as *IFIT5, EIF2AK2, APOBEC3G, APOBEC3F*, and others. Downregulated genes included *PRKN, EIF2AK4, RNASEL, APOBEC3C, BANF1*, and *MAVS* (Supplementary Table S1).

**Figure 13:**
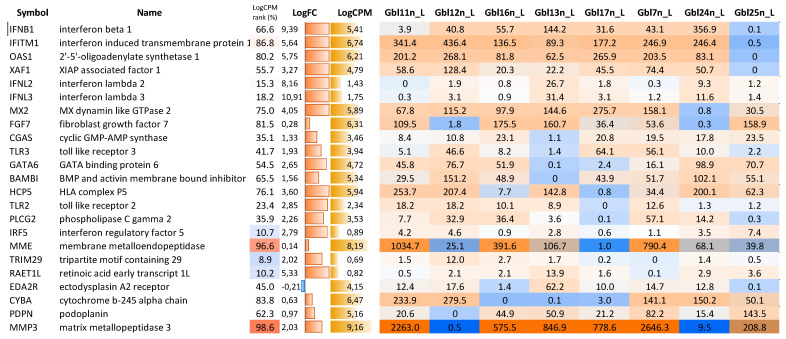
Mini heatmap (blue-to-red color logarithmic scale; from 16-fold downregulated to 16-fold upregulated relative to the average values) illustrating gene expression level in MV-infected GBM cells normalized to the average value (geometric mean). CPM tank (%)—position of a gene (0…100) in a list of all genes sorted by average expression level (read counts per million). LogFC—binary logarithm of expression level fold change.

However, cell lines responded to MV-infection with several notable differences. We observed that Gbl7n, Gbl11n, Gbl13n, Gbl16n, Gbl17n, Gbl24n, and Gbl25n lacked expression of several key factors of immune defense against MV-infection: Gbl25n—*IFNB1* and, thus, a wild spectrum of ISGs; Gbl11n—*IFNL2* and *IFNL3* (and also exhibited a low level of *IFNB1* expression); Gbl24n—*MX2*; Gbl13n—*CGAS* and *TLR3*; Gbl17n—*TLR2*, *PLCG2* and *IRF5*; Gbl7n—*TRIM29* and *RAET1L*; Gbl16n—*EDA2R* and *CYBA*; while Gbl12n (which was the most resistant to oncolysis by MV-infection) did not express sphingosine kinase 1 receptor 3 necessary to MV replication [24] (Figure 13).

**Figure 14:**
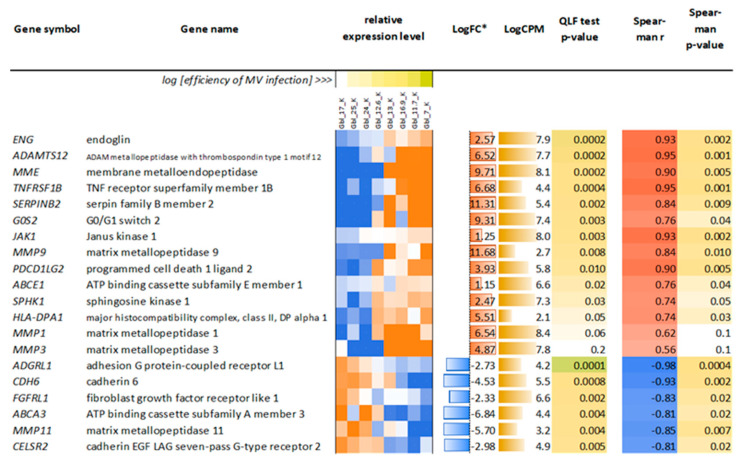
20 most remarkable genes whose expression in uninfected cells is associated with the efficiency of subsequent MV infection. * LogFC—binary logarithm of expression level fold change. Fold change is calculated by the slope of the line approximating the dependence of the expression of a particular gene on the MV infection efficiency across different cell lines. In the “ideal” case, when all the points of this dependence lie on the straight line, fold change is calculated as the ratio of the expression level in the cell line with the highest infection efficiency (Gbl7n) to the expression level in the cell line with the lowest efficiency (Gbl17n). QLF—quasi-likelihood F-test. Relative Expression level —mini heatmaps illustrating per-sample gene expression profiles normalized to the average value (geometric mean) across all samples, per each gene (blue-to-red color logarithmic scale; from 16-fold downregulated to 16-fold upregulated relative to the average values).

Then, to identify factors that determine susceptibility to MV, the correlation between gene expression patterns in uninfected cells and the efficiency of MV infection was performed by ranking cells according to the rate of primary infection as presented in Figure 6 (range of columns at 18 h.p.i.). The analysis of a list of all genes sorted by expression level in ranked cell lines by MV entry allowed us to identify genes with the highest coefficient of correlation (r) that could determine and predict sensitivity to the virus (Figure 14). Such potential genes were *ADAMTS12*, *MME, JAK1, MMPs, ABCE1, SPHK1*, and others that could facilitate MV entry.

**Figure 15:**
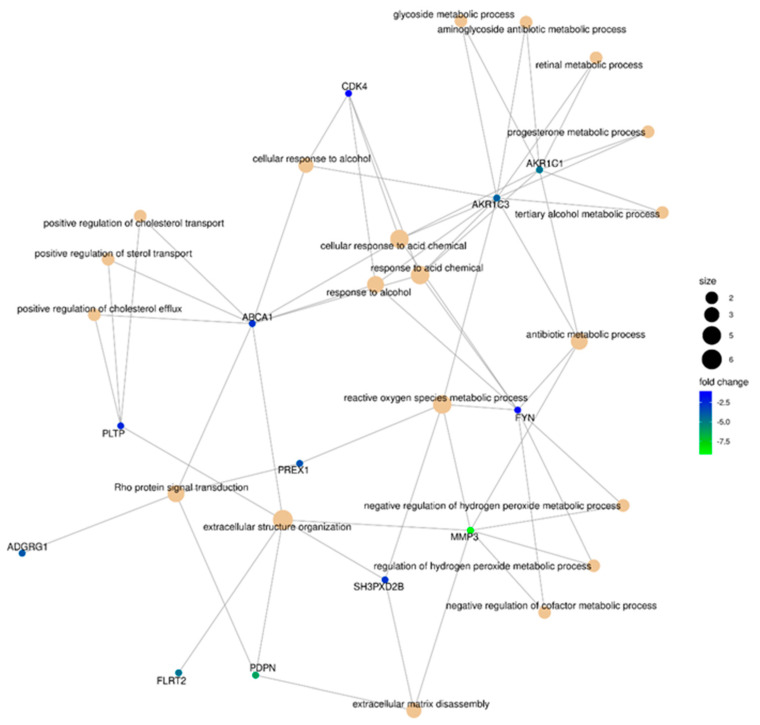
Gene-concept network for top 40 genes downregulated genes in MV-resistant cells, Gbl12n (both before and after infection, joint analysis). “Size” is proportional to the log number of genes involved in the current GO term.

**Figure 16:**
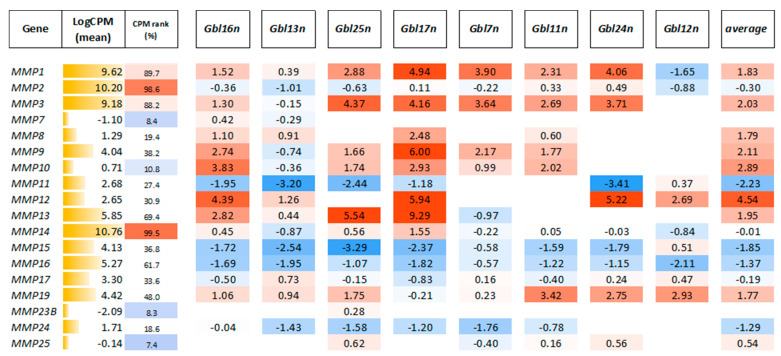
MMPs expression level in infected compared to uninfected GBM cells. Values represent LogFC (fold change) for each cell line. Empty cells mean the expression of an individual MMP transcript below the detectable level. CPM—counts per million reads.

In order to identify candidate genes that may control the susceptibility of GBM cells to MV L-16 infection and oncolysis, transcriptional profiles between sensitive cells and resistant cell lines were compared. The GO terms analysis of sensitive cells and resistant Gbl12n cell lines allowed us to identify *ADGRF5* as the most upregulated gene and *MMP3* as the most downregulated differentially affected gene (Figure 15). Furthermore, a closer look at the MMP’s expression profile revealed that the most abundant transcripts in infected GBM cells were *MMP1, MMP2, MMP3,* and *MMP14*, while the *MMP3* expression level correlated with sensitivity to MV-induced oncolysis in all cell line tested (particularly in Gbl12n it was not detected) highlighting the potential role of MMPs in productive replication of MV L-16 (Figure 16).

Altogether, these results prove that GBM cell lines respond to MV-infection by changes in their transcriptomic response and response to virus infection is also observed in cells that failed to produce IFN-I, such as Gbl25n cells.

We observed diversity among GBM cells in regard to their ability to express and respond to type I IFN pathway induction indicating the difference in the response to MV infection by all GBM cell lines tested. However, none of these cell lines displayed induction of the total gene set necessary for effective and complete inhibition of virus replication.

### 3.5. GBM Cells Release Inflammatory Cytokines after Infection with MV L-16

Cytokines may play a critical role in contributing to the complexity and lethality of GBM. Cytokines expressed by GBM cells may have a wide variety of influences including tumor cell invasion, proliferation, migration, neoangiogenesis, and immune cell infiltration. While some cytokines may support tumor growth, others may inhibit it, and some may have dual functions.

**Figure 17:**
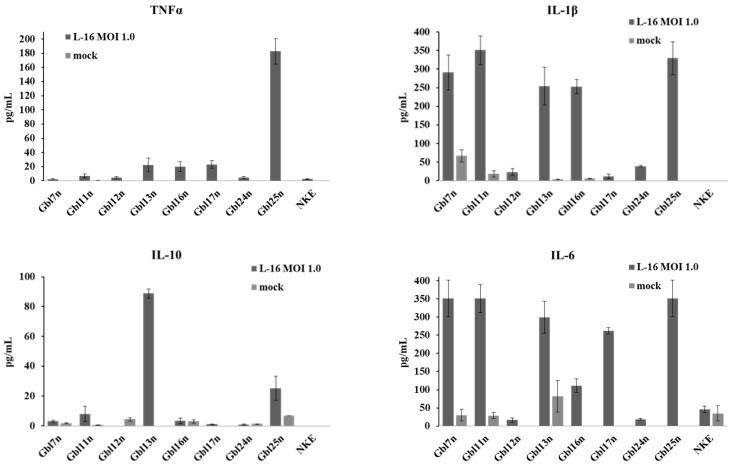
Products of pro- and anti-inflammatory cytokines by GBM cells. The supernatants of MV-infected and mock-infected cells were collected 48 h p.i. The levels of TNFα, IL-1β, IL-10, and IL6 were determined by the ELISA. Columns represent the average values of three independent experiments; error bands indicate SD.

As shown above, the GO term analysis revealed that biological processes involved in cytokine production, regulation, and processing are enriched in MV-infected GBM cells. To verify the up-regulation of the cytokine production on the protein level, supernatants collected from cells infected with varying MOI of MV were analyzed by ELISA for the presence of pro- and anti-inflammatory cytokines—TNFα, IL-1β, IL-6, and IL-10at 48 h p.i. (Figure 17).

Among nine cell lines tested, Gbl13n, Gbl16n, Gbl17n, and Gbl25n cells produced a tumor necrosis factor, TNFα, at a significant level in response to MV infection, while no constitutive production was detected. TNFα is known to have dual effects on the tumor microenvironment. The antitumor effects of TNFα include its ability to stimulate T-cell growth and enhance monocyte, granulocyte, and natural killer cell cytotoxicity, at the same time TNFα secretion leads to the promotion of glioma formation and development through angiogenesis [25]. All GBM cells, except for Gbl17n, responded to MV-infection with IL-1β production, although the level of production displayed large variation: the level in Gbl7n, Gbl11n, Gbl13n, Gbl16n, and Gbl25n cells were 10-fold higher than in Gbl12n, and Gbl24n. Consistent with data from transcriptome analysis Gbl7n, Gbl11n, Gbl13n, Gbl17n, and Gbl25n cells also largely expressed IL-6 pro-inflammatory cytokine in response to MV, while the infection exerted significantly lower levels of IL-6 production in Gbl12n, Gbl16n, and Gbl24n cells. In addition, a constitutive IL-6 production in Gbl7n, Gbl11n, and Gbl13n was also observed. Finally, for Gbl13n and Gbl25n cell lines, the high levels of production of anti-inflammatory cytokine IL-10, a well-known regulator of the activity of NF-κB and the JAK-STAT signaling pathway, were detected. Interestingly, the infection of the Gbl12n cells reduced IL-10 release relative to uninfected cells. In contrast to GBM cells, no cytokine production was observed in NKE cells in response to MV-infection.

Taken together, all analyzed GBM cells responded to the MV infection with cytokine production. The cytokine production correlated with the corresponding gene expression level. However, as for the transcriptome analysis, no common pattern of cytokine production in GBM cells was revealed.

## 4. Discussion

GBM is the deadliest form of brain cancer, with a median survival of less than 2 years despite surgical resection, radiation, and chemotherapy. GBM’s rapid progression, resistance to therapy, and inexorable recurrence have been attributed to several factors, including its rapid growth rate, its molecular heterogeneity, its propensity to infiltrate vital brain structures, the regenerative capacity of treatment-resistant CSMs, and challenges in achieving high concentrations of chemotherapeutic agents in the central nervous system [26]. Immunotherapy is being actively pursued for GBM because of its potential to overcome the challenges. OVs can overcome the immunosuppressive microenvironment of tumors due to durable responses elicited by the introduction of high-quality neoantigens. Thus, live attenuated MV vaccine strains have recently been shown as promising therapeutic agents against human malignant cells. Previous studies using the Edmonston-B strain of MV have evidenced clinical benefits for the treatment of GBM [27].

In this study, the oncolytic potential of the Russian vaccine strain of MV, L-16, was investigated against human GBM cells. Eight primary GBM cell lines were established and preliminarily characterized: half of these carried mutations in the *TP53* gene, while three (Gbl13n, Gbl16n, and Gbl25n) carried mutations both in *TP53* and *PTEN* genes. These defects are typical for GMB cells as tumor suppressor genes, such as genes encoding for p53, p21, p16, and PTEN are commonly mutated in GBMs [28]. A previous study described that human GBM stem cells with patient-specific p53 mutants and p53-Ser15 phosphorylation are selective targets for parvovirus [29]. Another study assessed the viral permissiveness of myeloma cells to the oncolytic MV in relation to *TP53* status. It was shown that the p53 pathway regulated CD46 expression and viral infection in primary myeloma cells, highlighting an increased sensitivity in p53-deficient cells. However, the correlation was not strict, suggesting that additional regulations are involved in viral sensitivity [30]. We demonstrated that almost all these established GBM cell lines, p53-deficient as well as p53-competent, were susceptible to productive infection by MV; the only exception was Gbl12n, which was considered largely resistant to MV infection. It should be noted that the same cell line was characterized by a slow proliferating rate while other GBM cell lines had doubling times comparable with the control cell line.

Despite the different history of cell culture adaptation of wild-type MVs, the L-16 strain, similar to the MV Edmonston-B strain, preferentially uses CD46 as an attachment receptor. Furthermore, the hyperexpression of the CD46 on the surface of tumor cells allows the MV to selectively infect and lyse the tumor [22]. All GBM cell lines tested expressed CD46, as did non-tumor cell lines. Though some variation of CD46 expression was observed, our data indicate that the CD46 receptor expression is an important but not essential mechanism, and other factors should contribute to the varied effects observed upon MV infection. Thus, tumor-related abnormalities in the regulation of mRNA translation suppressing IFN-induced inhibition of cell proliferation and apoptotic signaling could also facilitate selective replication of viruses in tumor cells [31].

In other studies, the presence of an intact type I IFN-response pathway in patient tumor samples was shown to correlate strongly with the suppression of MV replication in these cells. The type-I IFN gene expression level observed in the healthy cells and primary tumor cells resistant to virus-mediated oncolysis indicated that the cell that retained functional antiviral type-I IFN pathways are capable of suppressing the replication of MV. In contrast, cells sensitive to MV infection have been found to be defective in these pathways [32,33,34,35]. Thus, in this study, post-entry events in GBM cells in response to viral infection with MV strain L-16 were analyzed by RNA-sequencing in an attempt to determine genes mediating the sensitivity of GBM cells to oncolytic MV.

Transcriptome analysis by RNA-sequencing provides a valuable tool to comprehensively examine and identify pathways that are affected by virus infection. Analyzing gene expression in response to MV infection in GBM cells by transcriptome analysis, we also demonstrated that, despite the induction of IFN-β gene expression and interferon-stimulated genes (ISGs), GBM cell lines were still permissive for MV infection, albeit their sensitivity to MV induced oncolysis was different.

Furthermore, the significantly differentially expressed genes between treated and untreated GBM cells were subjected to pathway enrichment analyses. The significant groups with numerous genes for the GO biological processes annotation were identified, such as defense response to a virus, constitutive activation of the type-I IFN singling, negative regulation of viral genome replication, and other processes. Comparing up- and downregulated genes in the infected and uninfected GBM cells using GO term analysis allows us to identify differences in biological processes and pathways between sensitive and resistant cells providing new insights into the molecular mechanisms involved. Overall, our data showed that glioblastoma cells varied in regard to their ability to produce and respond to type I IFN.

Previous transcriptional profiling analysis following MV infection of primary human GBM cells, as well as control cell lines, revealed that MV induces a robust IFN-stimulated gene response. Furthermore, these studies allowed us to identify defects in IFN response responsible for the sensitivity of these cells to MV infection, such as *IFITM1* and *RSAD2* [34,35,36,37], as well as the correlation of effective paramyxoviral reproduction with a high abundance of *ABCE1* [38]. Other studies reported the frequent homozygous deletions of type-I IFN genes (IFN-β and IFN-α) responsible for cancer cell sensitivity to oncolytic MV [39]. However, in our study, we observed a completely reversed landscape of transcript expression: mRNAs of *IFITM1*, *RSAD2*, and *IFNB1* genes were well presented almost in all GBM cells studied, except for Gbl25n defective for type I IFNs and subsequent ISGs (Supplementary Table S2). These differences could rely on the use of distinct remote strains of MV.

To identify factors that determine susceptibility to MV the correlation between the efficiency of MV infection and gene expression patterns in uninfected cells was performed by ranking these cells according to the rate of primary infection. The analysis of the list of genes in ranked cells by MV entry allowed us to identify genes that could potentially determine and predict sensitivity to the virus. Among such genes were *MME, ABCE1, MMP11, MMP9, MMP1, JAK1, SPHK1*, and others playing role in MV infection as reported above. Interestingly, *MME, MMP2, MMP3, MMP7, MMP12, ADAM9*, and *ADAM12* were known to be upregulated in radioresistant GBMs, while sphingosine kinase 1 (SPHK1) phosphorylates sphingosine to produce sphingosine-1-phosphate, which promotes GBM invasiveness [40] and, on the other hand, overexpression of sphingosine kinase 1 enhances MV replication [41]. Additionally, such a study allowed us to reveal the importance of *ABCE1* in MV entry. Together, we hypothesize that multiple factors differentially expressed in non-tumor and tumor cells could contribute to efficient MV replication in GBM cells.

Furthermore, the GO terms analysis of sensitive cells and resistant Gbl12n cell lines allowed us to identify the *MMP3* gene as the most significantly differentially regulated gene. *MMP3* is a generally upregulated gene; however, in Gbl12n cells, its expression was absent in response to MV infection. In previous studies, it was shown that the fusion protein of MV can be selectively activated by tumor-secreted MMPs [42]. As mentioned above, tumor cells can be characterized by the expression of specific proteins, such as CD46 molecules, and their invasive growth is facilitated by tumor-specific proteases. MMPs are overexpressed in a wide variety of human tumors to facilitate the invasive growth of the tumor by degrading the extracellular matrix surrounding the tumor nodules. Multiple MMP family members, such as collagenase 1 (MMP-1), gelatinase A (MMP-2), gelatinase B (MMP-9), matrilysin (MMP-7), and membrane-type (MT)-MMPs, have been correlated with tumor progression such as the formation of metastasis and bad prognosis [43,44]. There is ample evidence suggesting a correlation between specific MMPs and glioma progression [45]. Thus, oncolytic MV takes advantage of these properties by rendering proteolytic activation of the fusion protein by specific tumor-associated proteases. A previous study underlined the clinical potency of the MMP activation concept, particularly MMP-2, as a strategy to generate safer oncolytic viruses for the treatment of primary and secondary liver cancers [46] and GBM without loss of potency [47]. However, different strains may require different tumor-associated MMPs for MV activation.

Thus, our data demonstrate innate variations in IFN response antiviral defense mechanisms within tumors that can have a statistically and biologically significant impact on MV replication. Therefore, our pilot study indicating the effective elimination of primary GBM cells by L-16 of MV in vitro could contribute to the development of approaches for the treatment of GBM based on the L-16 strain as a platform for further engineering and could complement the currently available approaches to drug therapy due to the selectivity of viral infection and spreading in tumor tissue with minimal toxic effects on surrounding normal cells.

## Figures and Tables

**Table 1:** Glioblastoma cell lines origin.

Primary Cell Line	Patient (Sex, Age)	Diagnosis, WHO Grade	Gene	Mutation
Gbl 7n	♂ 56	Glioblastoma (GIV)	-	-
Gbl 11n	♀ 58	Glioblastoma (GIV), primary multiple metachronous tumors	*PREX2*	c.778_779ins
Gbl 12n	♂ 57	Glioblastoma (GIII-GIV), relapse	*AR* *ATM*	c.234_239dup c.8734A > G
Gbl 13n	♂ 45	Glioblastoma, relapse	*PTEN* *MRE11* *TP53*	c.T821C c.579_582del c.C460T
Gbl 16n	♂ 47	Glioblastoma, relapse	*TP53* *PTEN* *TAF1L*	c.A419G c.A588T c.C2746T
Gbl 17n	♂ 68	Glioblastoma (GIV)	*PTEN* *ERBB3* *PIK3CG* *TOP1*	c.1524_1525ins c.C2150T c.A766T c.533_538del
Gbl 24n	♂ 49	Giant cell glioblastoma (GIV)	*PIM1*	c. * 104A > G
Gbl 25n	♀ 70	Glioblastoma (GIV)	*PTEN* *TP53*	c.635-1G > A c.C263T

(*) means nucleotide position in the three prime untranslated region (3’-UTR)

## Data Availability

Not applicable.

## Supplementary Materials

The following supporting information can be downloaded at: https://www.mdpi.com/article/10.3390/v14112433/s1, Table S1: GO Terms (BP) topGO enrichment; Table S2: DE results, Log.rel.CPM profiles-Resist vs Sensit [L16]; Figure S1: Immunofluorescence analysis of Gbl11n (upper panels), Gbl13n (middle panels), and Gbl24n (lower panels) cells. a—staining for nestin, b—nuclei counterstained with DAPI, c—merge. Scale bar—10 µM. 7 × 10^4^ cells were seeded into a cell culture dish (d = 10 cm) with glass slides in 1 mL of medium and incubated in at 37 °C with 5% CO_2_ overnight. Thereafter the culture medium was discarded and cells were washed twice with ice-cold PBS. Then 1 mL pre-cooled paraformaldehyde (4%) was added to the glass slides and kept for 10 min at 4 °C to fix the cells. Next, cells were permeabilized by treatment with 0.25% Triton X-100 for 5–10 min at room temperature. In the next stage, nestin antibody (Abcam, UK) was diluted in PBS at a 1:50 dilution rate and added dropwise to the cells. The glass slides were then incubated for 1h in dark at room temperature, then washed three times with PBS. Next, secondary antibody was added after being diluted with PBS at a 1:600 dilution rate, incubated for 45 min in dark at room temperature. Glass slides were placed on a microscope slide and sealed with elvanol. The sealed samples were kept overnight in dark at room temperature, and then their images were taken using a fluorescence microscope (Zeiss Axioplan 2, Switzerland). The immunofluorescence intensity for all groups was measured using ImageJ software.
