# Supplementary figures and images for: Transcriptome Analysis of Human Glioblastoma Cells Susceptible to Infection with the Leningrad-16 Vaccine Strain of Measles Virus
Source: Viruses. 2022 Nov 2;14(11):2433. doi: 10.3390/v14112433 (PMC9696624; doi:10.3390/v14112433)

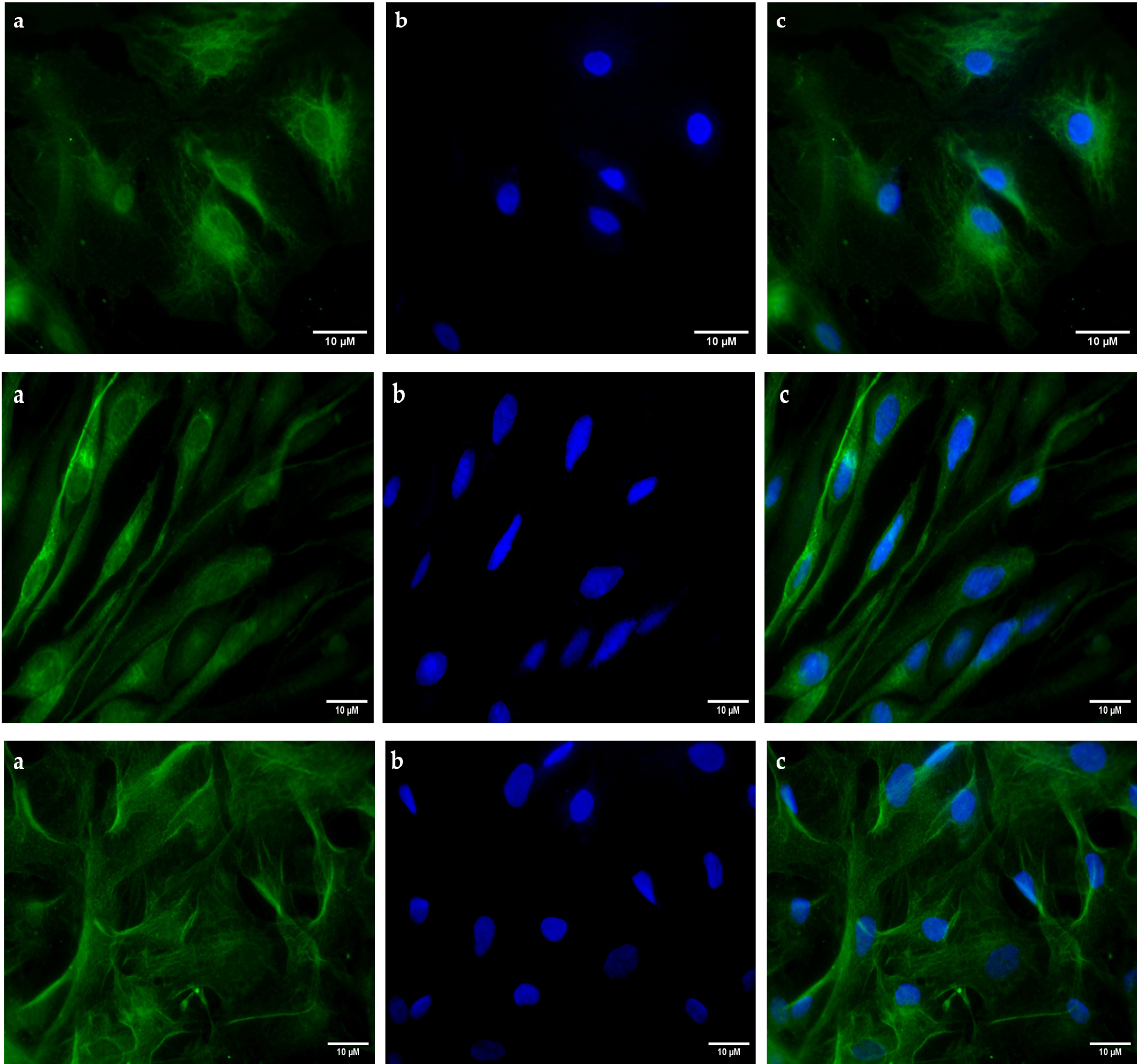

Supplement: Supplementary file 1 [file viruses-14-02433-s001.zip › FigureS1.png]
